# Multi-Sensor Wearable Health Device Framework for Real-Time Monitoring of Elderly Patients Using a Mobile Application and High-Resolution Parameter Estimation

**DOI:** 10.3389/fnhum.2021.750591

**Published:** 2022-01-17

**Authors:** Gabriel P. M. Pinheiro, Ricardo K. Miranda, Bruno J. G. Praciano, Giovanni A. Santos, Fábio L. L. Mendonça, Elnaz Javidi, João Paulo Javidi da Costa, Rafael T. de Sousa

**Affiliations:** ^1^Department of Mechanical Engineering, University of Brasília, Brasília, Brazil; ^2^Department of Electrical Engineering, University of Brasília, Brasília, Brazil; ^3^Department 2-Campus Lippstadt, Hamm-Lippstadt University of Applied Sciences, Hamm, Germany

**Keywords:** embedded high-resolution parameter estimation, healthcare multi-sensor wearable hardware development, health monitoring application architecture, ESPRIT, photoplethysmography

## Abstract

Automatized scalable healthcare support solutions allow real-time 24/7 health monitoring of patients, prioritizing medical treatment according to health conditions, reducing medical appointments in clinics and hospitals, and enabling easy exchange of information among healthcare professionals. With recent health safety guidelines due to the COVID-19 pandemic, protecting the elderly has become imperative. However, state-of-the-art health wearable device platforms present limitations in hardware, parameter estimation algorithms, and software architecture. This paper proposes a complete framework for health systems composed of multi-sensor wearable health devices (MWHD), high-resolution parameter estimation, and real-time monitoring applications. The framework is appropriate for real-time monitoring of elderly patients' health without physical contact with healthcare professionals, maintaining safety standards. The hardware includes sensors for monitoring steps, pulse oximetry, heart rate (HR), and temperature using low-power wireless communication. In terms of parameter estimation, the embedded circuit uses high-resolution signal processing algorithms that result in an improved measure of the HR. The proposed high-resolution signal processing-based approach outperforms state-of-the-art HR estimation measurements using the photoplethysmography (PPG) sensor.

## 1. Introduction

Nowadays, health systems, including hospitals and their intensive care units (ICU), are challenged by a substantial need to increase critical care capacity due to the Coronavirus Disease 2019 (COVID-19) pandemic (Phua et al., 2020). This same paper highlights the importance of streamlining workflows for rapid diagnosis and isolation, clinical management, and infection prevention essential to caring for COVID-19 patients. It also emphasizes the need to protect healthcare workers and other patients while supporting ICU practitioners' activities, hospital administrators, governments, and policymakers. As elderly people are more vulnerable to COVID-19, according to Rezende et al. (2020), and other diseases, it is important to ensure continuing healthcare to this part of the population using no-contact methods.

As suggested in Alwashmi (2020), health systems invest in automatized and scalable digital health support solutions, such as healthcare wearable devices and information systems empowered with artificial intelligence. Such automatized digital health solutions allow real-time 24/7 health monitoring of patients, prioritizing medical treatment according to the patients' health conditions, reducing medical appointments in clinics and hospitals, by sharing secure information among healthcare professionals. This is especially of interest when treating the elderly, as they are at greater risk in hospital environments as concluded in Costantino et al. (2021). Therefore, the framework presented in this paper can enable fewer hospital admissions of elderly people while closely monitoring their health and prioritizing more severe cases.

According to Grand View Research (2020), the global market for wearable medical devices was valued at USD 13 billion in 2019, with an expected annual growth rate of 27.9% until 2027. Still, according to Grand View Research (2020), multi-sensor health wearable devices are becoming popular due to the cost reduction of remote health monitoring technologies, including home healthcare. Moreover, an affordable and precise health-centered wearable device can allow for novel monitoring infrastructure and increase patients' quality of life.

In this paper, we propose a multi-sensor wearable health device (MWHD) framework with a real-time monitoring application and high-resolution parameter estimation. The proposed hardware includes sensors for step counting, pulse oximetry, heart rate (HR), and temperature measurements. Since wireless communications require a significant consumption of device battery energy (de Freitas et al., 2012; Marinho et al., 2013), the proposed MWHD optimizes the battery usage by using Bluetooth Low Energy (BLE). In terms of parameter estimation, the embedded integrated circuit programmed with high-resolution signal processing algorithms processes the sensors' signals, allowing improved analysis of the steps, pulse oximetry, and HR. Finally, the patient's medical information is reliably provided by the real-time monitoring application to healthcare workers.

This paper is composed of five sections, including this introduction as section 1. Section 2 shows related works in health data signal processing and healthcare platforms. Section 3 proposes a wearable device prototype using photoplethysmography (PPG), an algorithm for HR estimation, and a software platform architecture for remote healthcare supervision. Section 4 presents the methodology and results of the performance comparison of PPG processing algorithms for HR estimation. Finally, section 5 concludes the paper.

## 2. State of the Art

In Wu et al. (2020), the feasibility of a compact wearable sensor patch for measurements of different physiological signals, including PPG and body temperature, is presented. The wearable sensor system transmits the physiological measurements wirelessly to a gateway using a BLE module. The health data is encrypted, stored, and analyzed on the Internet cloud.

Concerning the usefulness and acceptance of wearable devices among the elderly population, paper (Kekade et al., 2018) presents a systematic review and survey results. The authors show that fewer elderly people are using wearable devices, while more than 60% of them were interested in using such devices. This presents an opportunity to expand the adoption of wearables in elderly healthcare. Also, only 26% percent of individuals were willing to pay for a wearable device, showing a need to ally their use with healthcare systems and not necessarily personal use. Finally, despite the disadvantages of wearables, the paper found good compliance prospects and prescribed raising awareness of the technology.

In Puranik and Morales (2020), a digital filter for PPG signals collected from an MWHD is proposed using an adaptive neural network, allowing a more accurate estimate of the HR, resulting in a variation of 3% concerning the ground truth. In Chung et al. (2020), a deep learning approach is proposed for the HR estimation using PPG signals, achieving an absolute error of 1.5 beats-per-minute (BPM), outperforming state-of-the-art methods. Finally, the authors of Panwar et al. (2020) present a new deep learning model with the capability to estimate HR using only a single channel provided by the PPG signal, achieving an error for HR estimation of 0.046 BPM. Note that the usage of neural networks requires labeled data and the tuning of the neural network hyperparameters.

In Coffen et al. (2020), a new ring-shaped sensor is proposed to estimate the heartbeat using reflective PPG. The measurements are transmitted to a mobile phone via Bluetooth 4.0. Compared with the commercial solution, the ring-shaped sensor presented an error of 2% smaller.

Still, regarding form-factor preference and acceptance, authors of Kolasinska et al. (2018) assess the usability of a set of sensors hidden in everyday objects. When prompted which pieces of jewelry were more frequently used, seniors gave the two most common answers: a watch (39%) or a bracelet (39%). Besides, health practitioners found greater functionality in writs-located devices. The paper also found other indications of the willingness of elderly people to use such wearable devices.

In Wang et al. (2019), it improves the HR estimation by using a new notch filter, and the noise cancellation approach is based on the least mean squares. As a result, the error of the HR estimation employing this approach is smaller than 1 BPM using measurements from intensive physical activities.

Reference (Xiong et al., 2017) discusses challenges in HR estimation from a wrist band with PPG collected during intense exercises. First, Principal Component Analysis (PCA) and adaptive filtering are used for removing noise from the PPG signals. Then, to estimate the HR, a Support Vectors Machine (SVM) based approach is considered. The approach in Xiong et al. (2017) presents errors of 1.01 BPM.

In Przybyło (2019), a new method is proposed to estimate HR using PPG. The method also includes Blind Source Separation (BSS) to improve the results further. The achieved Root-Mean-Square Error (RMSE) is 6.1 BPM.

In Godfrey (2017), authors discuss limitations shown in commercial wearable devices, citing the main limitations as non-clinically oriented devices and failure to provide transparent functionality on account of proprietary software. The authors propose that wearable devices can accommodate the needs of older adults while simultaneously monitoring gait due to their importance in checking aging-related pathologies. The paper (Godfrey, 2017) concludes by recommending the use of multiple and higher-resolution processing algorithms to overcome current limitations in gait assessment.

A novel health wearable platform for the real-time monitoring of accidents of elderly people is shown in Lampoltshammer et al. (2014). In terms of performance, the improved sensors in Lampoltshammer et al. (2014) present a longer battery lifetime, allowing their usage by elderly people for long periods.

Authors present another similar system in Durán-Vega et al. (2019). The presented framework is used in real-time monitoring of patients of nursing homes and comprises a wearable device, mobile application, and necessary middleware. The paper presents assessments for more convenient use of both the wearable and the application on the patients and of their caregivers. Our solution diverges by focusing on any clinical setting, employing a high-resolution processing algorithm aiming for clinical-appropriate data. Besides, our solution also looks to lower the cost of the device, even more, keeping it around $25 US.

### 2.1. State-of-The-Art Simplified Model for PPG Waveforms

The PPG waveform can be modeled as a pulsating quasi-periodic component attributed to synchronous cardiac changes in the blood volume with each heartbeat. A slowly varying low-frequency component superimposes this pulsating component, with various lower frequency components attributed to respiration, sympathetic nervous system activity, and thermoregulation (Allen, 2007). The PPG signal is sampled with a sampling rate of *f*_*s*_. Such sampled PPG waveform is modeled by:


(1) $$\begin{array}{l} x\left[n\right]=A\cos\left[2\pi fn+\theta \right]+r\left[n\right], \end{array}$$

where *A* is the PPG signal amplitude, *f* is the relative frequency of the PPG signal normalized by *f*_*s*_, θ is the phase of the PPG signal, and *r*[*n*] is a component that comprises the noise and artifacts present in the PPG signal. Note that, due to the HR variability (HRV) (Buccelletti et al., 2009) and the Inter-Beat Intervals (IBI), HR is a time variable. However, we assume a short estimation interval, such that the model in (1) can be applied.

HR detection can then be formulated as a frequency estimation problem. Thus, by measuring the frequency parameter *f* of a periodic heart signal, given in Hz, we convert the HR to the corresponding value in BPM, given by BPM = *f* · 60.

In the literature, common approaches for the HR estimation that are applied in embedded systems include Fast-Fourier Transform (FFT) based (Santamaria et al., 2000), autocorrelation (Proakis, 2013), zero-crossing detection (Zhang et al., 2008) and peak detection (Scholkmann et al., 2012). These approaches are summarized in Appendices A.1, A.2, A.3, and A.4, respectively.

## 3. Proposed Healthcare Platform Composed of Low-Cost Hardware, High-Resolution Parameter Estimation Algorithms, and Real-Time Monitoring Application Architecture

**Figure 1:**
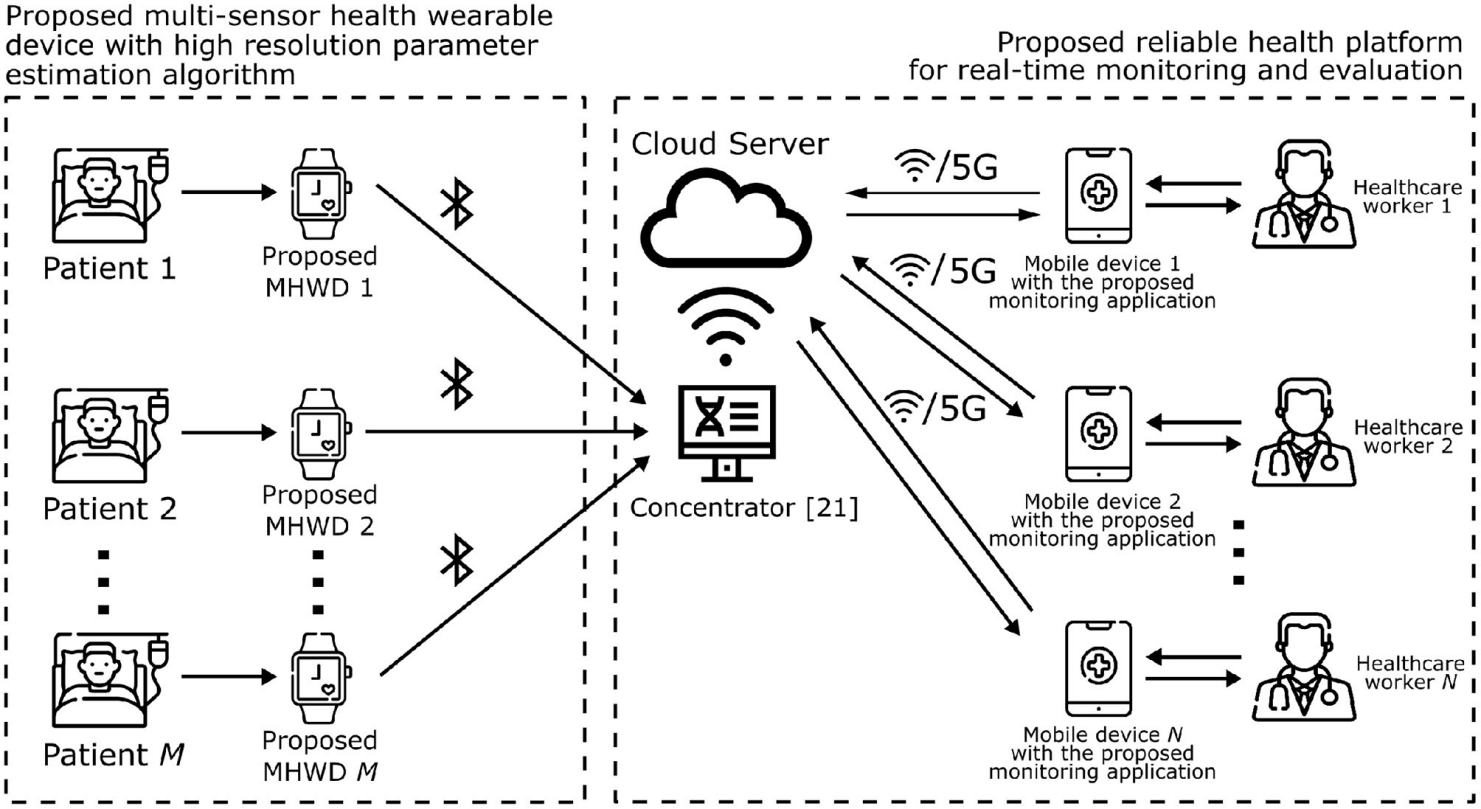
Diagram of the proposed healthcare framework, consisting of a multi-sensor health wearable device, PPG high resolution parameter estimation, and real-time monitoring application.

This section, as depicted in Figure 1 proposes the multi-sensor health wearable device framework with real-time monitoring application and high-resolution parameter estimation. According to Figure 1, each patient is assigned an MWHD, which gathers and processes each subject's health data. Patients can be isolated from each other and healthcare professionals, reducing contact to only the necessary to enforce health protocols. This approach better fits the needs for tighter healthcare procedures with seniors. The MWHD can transmit the processed data via a wireless communication protocol based on BLE to a concentrator device. Note that the concentrator hardware depicted in Figure 1 has been proposed in Prettz et al. (2017). This concentrator device uploads data to a cloud server, which interacts with the proposed monitoring application installed on mobile healthcare workers. The healthcare workers are thus able to monitor patients and collaborate in real-time.

This section is divided into three subsections. In section 3.1, we detail the proposed MWHD. In section 3.2, we propose the high-resolution processing algorithm for HR estimation. In section 3.3, we propose the healthcare platform for real-time monitoring.

### 3.1. Proposed Healthcare Wearable Device Including Multiple Sensors, Processor, and Low Energy Wireless Communication

**Figure 2:**
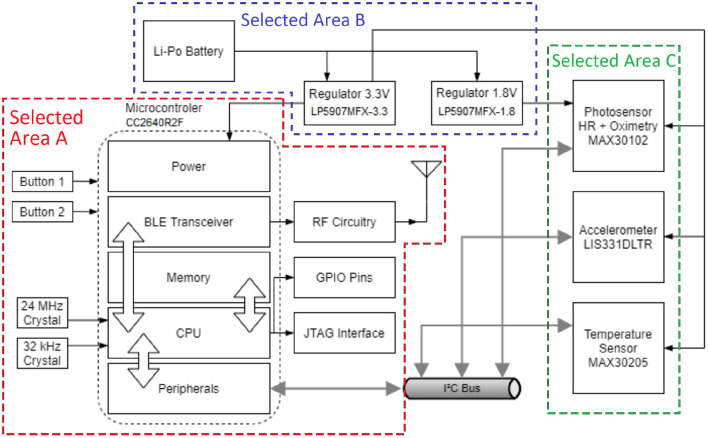
Block diagram showing the functional circuit components of the proposed prototype wristband.

The proposed MWHD prototype shown in Figure 2 is battery-operated and contains sensors to measure the health information of the user—namely HR, pulse oximetry, body temperature, and steps. The current paper focuses on high-resolution HR measurements. Additionally, an application of step counting is shown in Rega et al. (2019). We also implemented the other parameters as directed by the manufacturer's datasheet of the MAX30102 pulse oximetry sensor from Maxim Integrated (2018). The sensors communicate over an Inter-Integrated Circuit (I^2^C) bus with the microcontroller unit (MCU). The MCU then transmits data to mobile devices using BLE, thus minimizing power consumption.

Selected Area A of Figure 2 comprises the CC2640R2F Cortex®-M3 MCU circuit by Texas Instruments (2016) and additional components for operating the MCU and for the BLE communication interface. Two crystals are positioned to generate clock signals for different modes of the MCU, one with 24 MHz for regular speed operation and another with 32 kHz for low-power mode. The reduced speed during low-power mode saves power during idle operation. The General Purpose Input/Output (GPIO) pins and Joint Test Action Group (JTAG) Interface are made available in pin connectors for ease of access during testing of the MWHD prototype. The Radiofrequency (RF) circuitry is built according to the recommendations by Texas Instruments (2016) related to the 4 x 4 External Single-ended configuration, which requires a smaller board space and saves more power. Two push buttons are added for user input and interaction with the device.

Selected Area B of Figure 2 is the power sourcing part of the circuit from a small Lithium-Polymer (LiPo) battery. The nominal 3.7 V of the LiPo battery is regulated to 3.3 and 1.8 V using low-dropout regulators LP5907MFX-3.3 and LP5907MFX-1.8. The regulators guarantee a stable voltage of operation for the MCU and sensors during device operation and battery's charge and discharge cycles. In addition, the LiPo battery is equipped with a generic commercial micro USB recharging module with overcharge, over-discharge, and current protections.

Selected Area C of Figure 2 includes the sensors for health data measurements. The signals provided by the PPG sensor MAX30102 are used to estimate the HR and pulse oxymetry using red or infrared (IR) LEDs. In conjunction, red and IR LED signals enable pulse oximetry estimation based on the different absorption rates of arterial and venous blood. The prototype device also comprises an accelerometer LIS331DLTR, used for the step counter, to measure movement artifacts used for interference reduction on the PPG signal. For keeping track of body temperature variations, such as fever, the prototype utilizes a MAX30205 sensor. The sensor is positioned on the bottom side of the prototype device to contact the users' skin. The values read are calibrated according to the manufacturer's specifications in Maxim Integrated (2016).

The proposed printed circuit board (PCB) is designed as a 4-layer board to achieve routing requirements and reduce electromagnetic interference (EMI). Additionally, the proposed PCB is double-sided mounted, with a central processor and input buttons on the top and sensors on the bottom to contact the user's wrist skin (required by PPG and body temperature measurements). Test points are also positioned for power sourcing, communication interfaces, and JTAG debugging.

**Figure 3:**
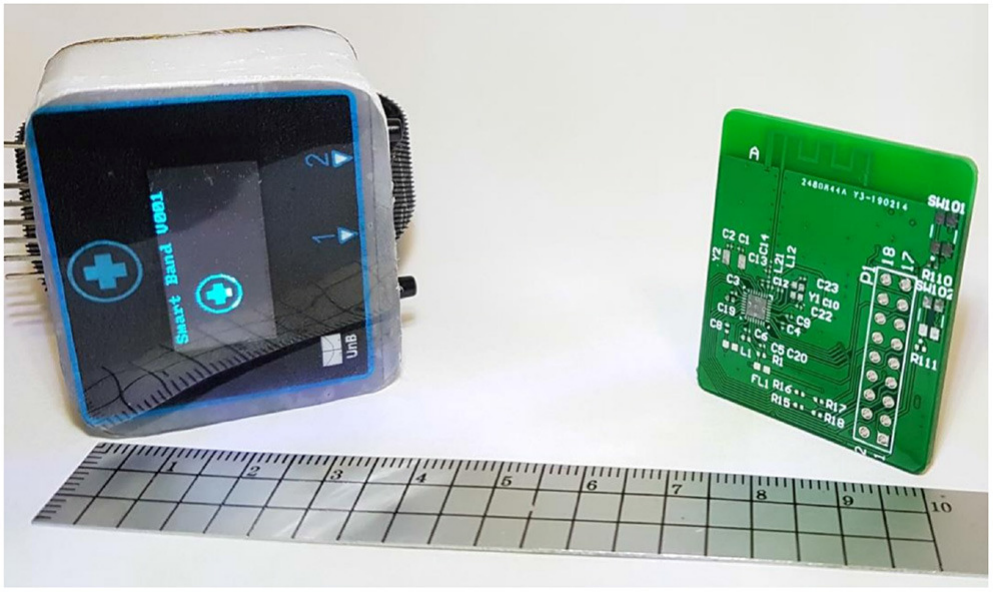
Dimensions of the PCB and enclosure produced for the proposed MWHD device prototype.

Figure 3 shows photos of the MWHD prototype produced according to the block diagram of Figure 2. On the left-hand side photo, the complete prototype encapsulated as a smartband is shown, while on the right-hand side photo, the PCB of the block diagram of Figure 2 is depicted.

The MWHD is projected as a low-cost device, enabling a cheaper complete healthcare solution to be deployed on a large scale. The cost of the components and PCB was USD 25.00 in a low volume run, indicating that bulk production can make further cost reductions. The final price of the device is on-par with other low-cost fitness wearable devices available in the market. This cost compares well with clinical health devices, which can cost more than USD 100.00 by a considerable margin.

We carried out the hardware prototype development and the proposed algorithm for HR estimation simultaneously. In addition, we gathered HR data from a MAX30100 device for experimental validation. This different PPG sensor module is equivalent to the projected prototype. The employed pulse oximeter and HR sensor integrated circuit captured data in peripheral oxygen saturation (SPO_2_) measuring mode, though pulse oximetry data is not presently considered. Furthermore, data from the IR LED from the sensor is considered during the tests due to reduced interference from ambient background lighting, while the red LED data is disregarded for the application.

The MAX30100 PPG sensor module communicates via I^2^C bus to the main MCU, which then transmits gathered data to the computer's serial port via a USB-to-Serial adapter. The experiment uses wired serial transmission to guarantee signal integrity further, avoiding adverse effects to any samples input to the algorithms due to events such as package drop or wireless interference.

### 3.2. Proposed High Resolution Signal Processing Algorithm for HR Estimation

A novel approach for HR detection applying the Estimation of Signal Parameters by Rotational Invariance Techniques (ESPRIT) (Roy and Kailath, 1989) algorithm is presented in this subsection. In Reis et al. (2017), the high-resolution signal processing technique named SPHINS is successfully applied for the frequency estimation in forensic applications. In Rega et al. (2019), a high accuracy step counter algorithm based on ESPRIT has been proposed using the accelerometer signals acquired by the sensors of our MWHD prototype proposed in section 3.1. Inspired by the outstanding results of Reis et al. (2017), Reis et al. (2016), and Rega et al. (2019), we propose the usage of the high-resolution signal processing algorithm ESPRIT to measure the HR.

The algorithm exploits the property of rotational invariance of signal subspaces spanned by two temporally displaced data sets (Roy and Kailath, 1989). A simplified description of the least-squared version of the ESPRIT algorithm is shown next based on Manolakis et al. (2005) and Reis et al. (2016).

By applying the Hilbert transform on the PPG waveform model in (1), we obtain the analytic representation of the signal *x*[*n*] ∈ ℝ given by:


(2) y[n]=x[n]+jℋ{x[n]},

where the operator H{} denotes the Discrete Hilbert Transform (DHT) and $j=\sqrt{-1}$.

We replace (1) into (2) and rewrite *y*[*n*] ∈ ℂ by taking into account that H{} is a linear operator, as:


(3) y[n]=Acos[2πfn+θ]+jℋ{Acos[2πfn+θ]}+r[n]+jℋ{r[n]}.

The signal *y*[*n*] can then be represented as a sum of the complex exponentials with added noise component *w*[*n*], comprising the real and imaginary parts of the noise component *r*[*n*]:


(4) $$\begin{array}{l} y\left[n\right]=A\exp\left[2\pi fn+\theta \right]+w\left[n\right]. \end{array}$$

By segmenting the samples obtained by *y*[*n*] in (4), we can build a data matrix **Y** where *N* is the amount of data records of the length-*P* time-window vector signal *y*[*n*], thus:


(5) $$Y={\left[y[0]\;y[1]\;\ldots \;y[N-1]\right]}^{\text{T}}\;\in \;{\mathbb{C}}^{P\times N},$$

where **y**[*n*] = [ŷ[*n*] ŷ[*n* + 1] … ŷ[*n* + *P* − 1]]^T^, and ^T^ is the transposition operator of matrices.

Next we compute the sample covariance matrix of (5) as follows:


(6) $$R_{y}=\frac{YY^{\text{H}}}{N}.$$

By applying the Eigenvalue Decomposition (EVD) in (6), we obtain the following expression:


(7) $$R_{y}=U\Sigma U^{\text{H}},$$

where **U** is an *P*×*P* matrix of right singular vectors and ^H^ denotes the Hermitian operator. Matrix **Σ** ∈ ℝ^*N*×*P*^ has dimensions *N*×*P* and is composed of singular values.

Matrix **U** can be decomposed as **U** = [**u**_*y*_0__|**U**_w_], where **u**_*y*_0__, the first column of **U**, is the vector that generates the signal subspace, of dimensions *P* × 1, formed by the singular vector corresponding to the maximum singular value of the data matrix **Y**. The remaining singular vectors form a matrix in which its columns correspond to the basis that generate the noise subspace **U**_w_, of dimensions *P*×(*P*−1), orthogonal to the signal subspace.

By writing vectors **u**_*u*_ and **u**_*d*_ formed by the first and last *P*−1 elements of **u**_*y*_0__, respectively, the rotational invariance presented previously, and exploited by ESPRIT, guarantees that:


(8) $$u_{u}\phi =u_{d},$$

where ϕ ∈ ℂ corresponds to the rotation scalar. By solving (8), the phase value estimation of ϕ is given by:


(9) $$\phi =∠\frac{u_{u}^{\text{H}}u_{d}}{u_{u}^{\text{H}}u_{u}},$$

where ∠ is the phase notation that denotes the phase angle value of the corresponding complex number.

We determine the frequency estimator $\hat{f}$ with the computed phase angle value of ϕ and the sampling frequency *f*_*s*_, thus:


(10) $$\begin{array}{l} \hat{f}=\frac{\phi }{2\pi }\cdot f_{s}. \end{array}$$

To estimate HR, we calculate the time window *T* of the measurement given by *T* = *n*_samples_/*f*_*s*_, that is equivalent to the duration of the measurement in seconds. Finally, the estimated value is $\text{BPM}=\hat{f}\cdot 60/T$.

Next, we present the summarized steps of the ESPRIT based algorithm for the HR estimation.

**Algorithm 1:** Proposed ESPRIT-based HR estimation via Hilbert Transform

Given signal *x*[*n*] in (1), sampled with frequency *f*_*s*_, during a time window of *T*:
1) Obtain signal *y*[*n*] according to (3) by applying the Discrete Hilbert Transform to *x*[*n*].
2) Segment the samples obtained in signal *y*[*n*] to obtain the data matrix **Y** in (5)
3) Compute the sample covariance matrix estimate ***R*_*y*_** of the data matrix **Y** as in (6).
4) Decompose ***R*_*y*_** in the corresponding EVD matrices to calculate by eigendecomposition matrix **U** according to (7), whose columns are the corresponding right eigenvectors of ***R*_*y*_**.
5) Determine the column **u**_*y*_0__ of matrix **U**, corresponding to the maximum singular value of data matrix **Y**.
6) Determine **u**_*u*_ and **u**_*d*_ by taking the first and last *P*−1 elements of vector **u**_*y*_0__.
7) Estimate the rotation scalar ϕ ∈ ℂ from vectors **u**_*u*_ and **u**_*d*_ based on the rotational invariance property as in (8).
8) Extract the estimated angle value of ϕ from (9).
9) Determine the frequency estimator using phase angle value of ϕ into (10).
10) Calculate the estimated BPM value equal to $\hat{f}\cdot 60/T$.

### 3.3. Healthcare Platform for Real-Time Monitoring and Evaluation

**Figure 4:**
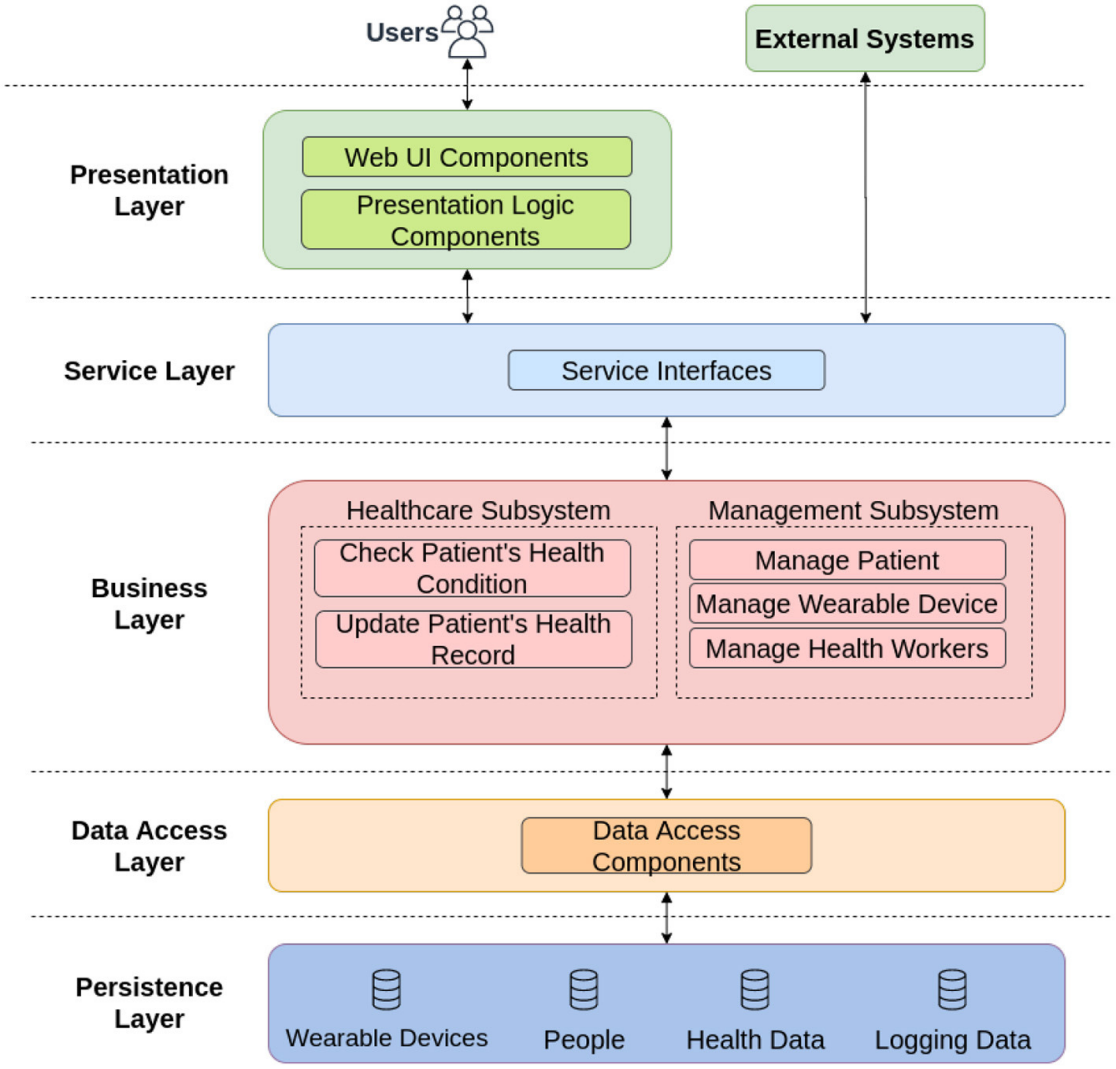
Software architecture for the developed real-time monitoring healthcare application.

The software architecture of the proposed application is divided into five layers, as depicted in Figure 4.

In the Presentation Layer of Figure 4, a login screen for authentication and registration of new users is defined. This feature allows users to register and identify their medical credentials and securely store their information in the application servers.

The Service Layer of Figure 4 exposes the business logic implemented in the software to potential consumers. One example of an external system is the concentrator, responsible for uploading data from wearable devices.

The Business Layer of Figure 4 is the logic behind the platform. We divided this layer into two blocks, in which the Healthcare Subsystem is used to check the data generated from the patient. The other block, called Management Subsystem, has an administrative function, tasked with supervising and managing multiple patients, wearable devices and healthcare workers.

The Data Access Layer of Figure 4 contains components to abstract the logic required to access the data stores. Such components provide common data access functionality, isolating the upper layers from the specific database technology, and making the application easier to maintain and configure.

The Persistence Layer of Figure 4 provides several advantages to the software since it is more efficient to save and retrieve data and provide for the whole application. In our context, we have four data sources, namely: Wearable Devices, People, Health Data, and Logging Data.

One advantage of the proposed online application in the solution framework is the interaction with the end-user. Using the platform, healthcare workers can track multiple users' health conditions in a centralized and reliable manner. Note that the proposed real-time monitoring platform can be integrated into other MWHD, as exemplified in Prettz et al. (2017), with the usage of a commercial MWHD.

Even though secure data communication is not a focus of this paper, it is essential to highlight that a distributed system such as this proposed healthcare application needs a fully distributed security system, such as proposed in Ferreira and de Sousa Jr. (2017). As it also comprises interactions according to the Internet of Things (IoT) paradigm and involves personal data, a particularly lightweight protocol for the authentication of devices, as proposed in de Almeida et al. (2018), is also paramount.

## 4. Experimental Validation

To evaluate the performance of the proposed ESPRIT-based HR estimation approach in section 3.2, measurements using the PPG sensor equivalent to that of section 3.1 are considered. Unfortunately, the MAX30100 sensor used for this experimental validation from Maxim Integrated (2014) has an analog-to-digital converter (ADC) with lower resolution than that available in the MAX30102 in Maxim Integrated (2018) sensor present in the MWHD prototype, and also different possible parameter configurations. Nevertheless, we believe the results presented here are extensible to the proposed MWHD due to equivalences in both sensors' PPG technology.

The experimental validation trials were performed by one voluntary person that was asked to perform specific activity levels to produce different heart rate observations for the measured values. The voluntary person wore a medical-grade PPG device—for ground truth—along with our proposed PPG measuring device. For each considered parameter, a total of five samples were captured, with a total duration of 10 s each. Initially, the voluntary was in rest, and then performed intense cardiovascular exercise for a defined time interval. Then, the voluntary rested after the cardiovascular exercise.

Initially, signal pre-processing is performed on the sampled data to eliminate artifacts and other detrimental factors that hinder each HR estimator's performance. Then, such processes are employed to account for signal processing present in PPG systems' real operation, maintaining this work's scope.

Motion artifacts are removed using an outlier detector that removes abruptly varying artifacts from the signal based on the derivative's high absolute values between consecutive samples. A low-frequency blocker filter—as proposed in Smith (2008)—is implemented with *R* = 0.95 to filter most of the low frequency noise in the signal. A 6-th order Butterworth low-pass filter with a cutoff frequency of 4 Hz is applied to attenuate high-frequency noise. It leaves an effective bandwidth that can detect an HR of up to 240 BPM.

To establish a referential target value for the real HR value, we employ a medical oximeter model ELERA SH-K3 that measures HR and pulse oximetry using red and infrared light for transmissive PPG, worn on the user's finger. Immediately before and after each measurement, the HR values of the device were logged. This gives an initial and final reference value for the oximeter in the measurement time frame, allowing for HR variation during the experiment to be taken into account. The values measured after the experiment are then used as ground truth values for calculating the RMSE of each estimator. As a result, we believe that the sensors' final readings better reflect the BPM values during the experiment, as they include the same time window of the sample measurements.

After processing the samples with the procedures described, the same PPG sample data points were input in each of the compared algorithms, generating the estimated heart frequency for each sample. These estimated values were then compared to the ground truth values for each sample. Results are expressed in terms of the RMSE, given by:


(11) $$\begin{array}{l} \text{RMSE}\left(f,\hat{f}\right)=\sqrt{\frac{1}{n_{\text{samples}}}\overset{n_{\text{samples}}-1}{\underset{i=0}{\sum }}{\left(f_{i}-{\hat{f}}_{i}\right)}^{2}}, \end{array}$$

Calculated for the estimator ${\hat{f}}_{i}$, referenced either from the oximeter's ground truth value *f*_*i*_ as read at the end of each measurement. After assessing each sample's individual RMSE, the general RMSE value for each algorithm was generated in each parameter's category. The performance comparison among the algorithms was based on the resulting general RMSE value for each algorithm in the categories considered.

The algorithms' scripts are developed in MATLAB version 2018a and were tested on Windows. Licensing is required for non-academic use of the software. The code used in this experimental validation is available on GitHub in Pinheiro (2021). Samples used are available on request.

**Figure 5:**
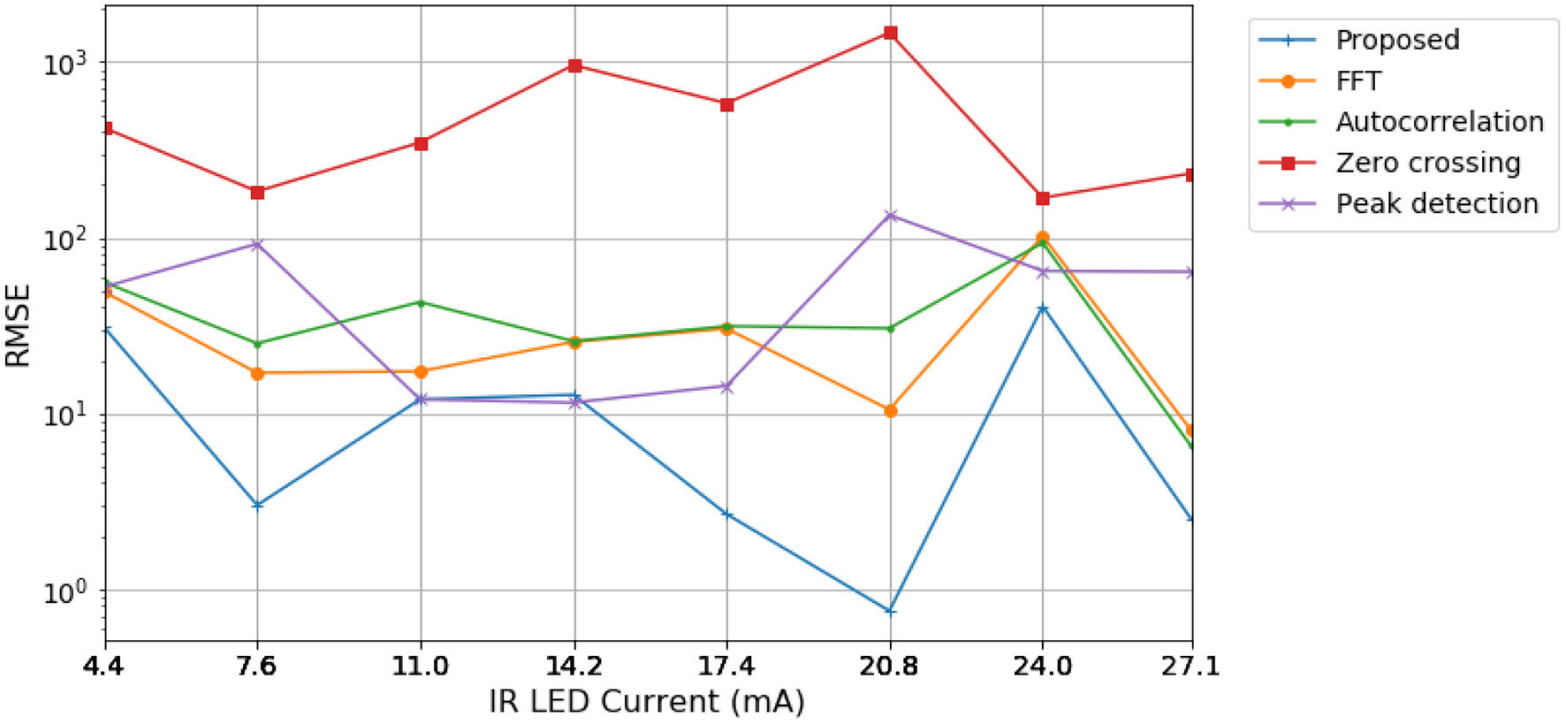
RMSE of estimated BPM values for each algorithm for different current level configurations, referenced to values read by the oximeter.

For experimental validation, the PPG sensor configurations vary to evaluate each algorithm's capabilities and robustness. Initially, the IR LED current level, which controls its transmission power, is investigated using the values made available on the sensor. Lower IR current levels decrease the device's energy consumption. However, as the LED power also decreases, the detected signal is weaker, which may increase the estimation error. Configurations from 30.6 to 50 mA saturated the sensor's ADC, generating no meaningful data. The remaining measurements were processed with a signal time window of 5 s and a sample rate of 100 Hz, amounting to 500 samples. In Figure 5, results are presented considering the oximeter reference, read right after experimental measurements ended.

According to Figure 5, the proposed algorithm based on ESPRIT resulted in lower RMSE values for most of the current values considered. Moreover, the proposed algorithm could perform more accurately at lower current levels, indicating saving more power.

**Figure 6:**
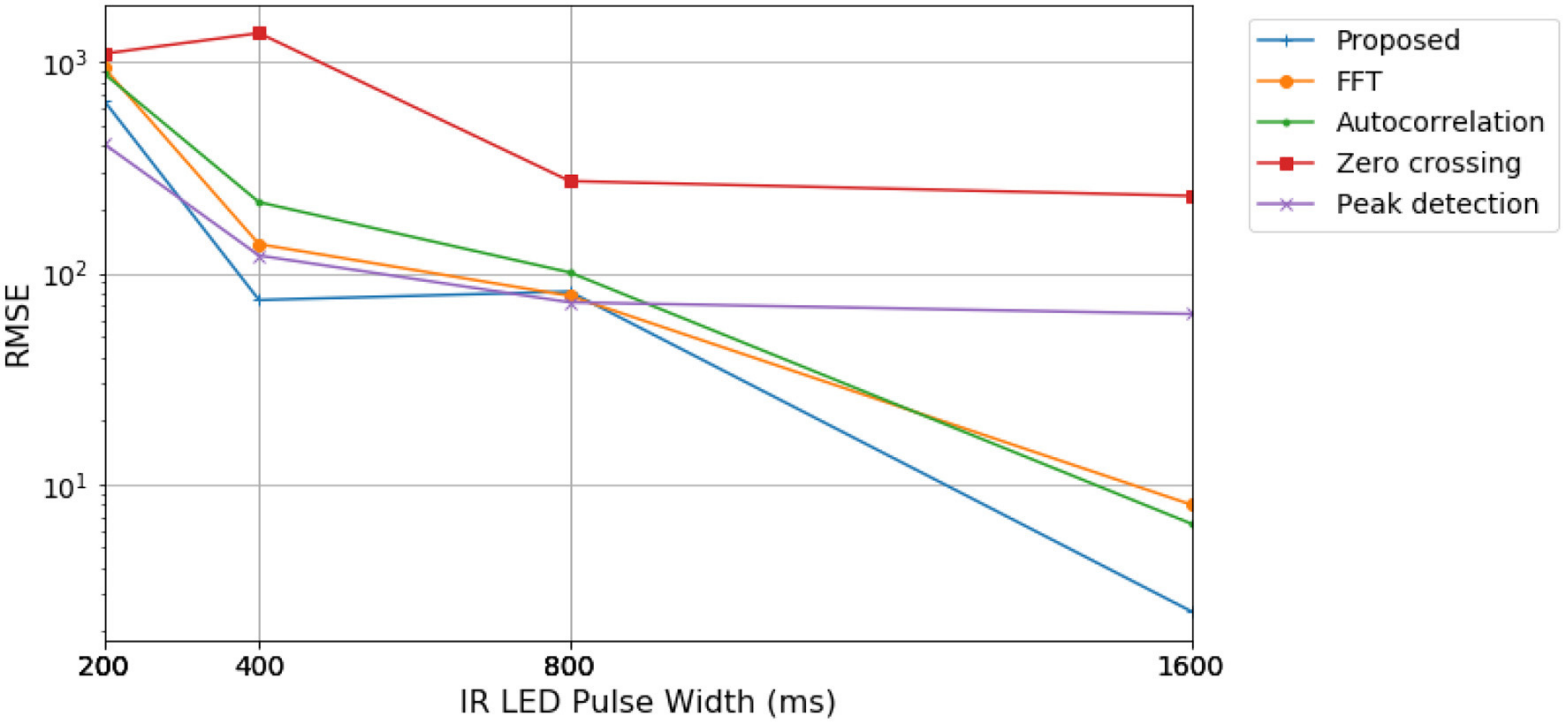
RMSE of estimated BPM values for different pulse width and corresponding ADC resolution configurations, referenced to values read by the oximeter.

In Figure 6, we varied the IR LED pulse width. Longer pulse widths increase power consumption and the detected signal's length, enabling the ADC to settle in a more precise value. Consequently, the ADC resolution available at the sensor is dependent on the selected pulse width, whose possible values are shown in Table 1. Experimental data was gathered, fixing the LED current level at 27.1 mA and sample rate at 100 Hz. Data were processed with 500 samples, corresponding to a 5 s time window. The RMSE of the estimations is presented by the final readings of the oximeter reference considered in Figure 6.

**Table 1:** LED pulse width configurations tested and the correspondent ADC resolution.

**Pulse width (μs)**	**ADC resolution (bits)**
1,600	16
800	15
400	14
200	13

The estimator results are consistent with the previous test case, with the proposed algorithm generally having lower RMSE values. These tests showed significant results regarding algorithm performance with reduced pulse width and ADC resolution. Pulse width shorter than 50% of the maximum value is shown to degrade estimation precision considerably. In addition, the shortest pulse width generated higher error values than other configurations. This may be due to signal ripple caused by pulse-width modulation (PWM) operation, which is more substantial the pulse is shorter. Another factor to consider is the lower ADC resolution corresponding to lower LED pulse width. Therefore, a lower sampling resolution can further impair the output PPG parameter estimation along with suspected ripple noise.

We also validate variations in the sample rate to corroborate with the previous experimental stages. A higher sampling rate decreases signal aliasing, yet the more massive samples per second require the ADC to settle in a lower resolution. The experimental results obtained show that higher sample rates have decreased precision in HR, possibly due to the imposed reduction in ADC resolution in each configuration tested, as can be seen in Table 2. Hence, a sample rate of 100 Hz is considered advantageous since it maintains a larger signal bandwidth and maximum ADC resolution.

**Table 2:** Maximum available ADC resolution for each sample rate configuration tested.

**Sample rate (Hz)**	**ADC resolution (bits)**
50	16
100	16
167	15
200	15
400	14

The proposed algorithm based on ESPRIT showed consistent results throughout the experiments performed. Moreover, it generally has lower RMSE values for lower IR LED current configurations and lower sampling frequencies, following the general trend in the pulse width variation. Hence, the increased precision demonstrates the potential for power-saving and more resilient performance in challenging scenarios.

An analysis of power consumption of the proposed MWHD was carried out in de Assis (2018) and is summarized here for further experimental validation. As a result, the current consumption was measured as 2.032 mA on average. That leads to a total battery autonomy of the MWHD of approximately 73 h, about a small-sized 150 mAh LiPo battery. Moreover, that enables the MWHD to be used for around 3 days without recharging, adding comfort to senior patients' use.

The connection between the MWHD and the real-time monitoring application is possible due to the Representational State Transfer (REST) protocol. We built an Application Programming Interface (API) to perform this connection with the database based on this protocol. We computed the times in different requests for the different pages of the platform. The results are presented in Table 3.

**Table 3:** Access times to different test requests performed to the API of the real-time monitoring application.

**Action**	**Access time (ms)**
Retrieve data from feed page	43.87
Access patient's profile	95.0
Add new patient	97.70
List teams	61.19
Request patients list	213.08
Add new healthcare worker	36.01

As shown in Table 3, the low latency of access validates that the proposed online platform can be used for real-time monitoring.

## 5. Conclusion

This paper proposes a multi-sensor wearable health device framework and a real-time monitoring application with high-resolution parameter estimation. A complete solution enables the needs of elderly patients to be better accommodated whilst ensuring that the latest COVID-19 related health protocols are observed. The proposed hardware includes step counting, pulse oximetry, HR, and temperature sensors. In addition, the proposed MWHD optimizes battery usage by using BLE. In terms of parameter estimation, the embedded system programmed with high-resolution algorithms processes signals from the multiple sensors used, allowing an improved estimation of the steps and HR. Finally, the patient's medical information is reliably provided to the healthcare workers by the real-time monitoring application.

Future works include a possible hardware modification to include an additional processor dedicated to data measurement and processing, ensuring that the proposed high-resolution ESPRIT algorithm runs alongside the BLE interface while conserving the most battery power. Furthermore, the proposed health application is considered to evolve with parallel and distributed processing based on microservices over GPU grids, thus allowing computing strategies to process multiple dataflows from devices for the sake of machine learning and pattern recognition. As mentioned, it will also leverage the exploitation of federated learning techniques over dataflows from multiple MWHD, thus allowing monitoring and artificial reasoning on grouped data from patients under supervision.

## Data Availability Statement

The raw data supporting the conclusions of this article will be made available by the authors, without undue reservation.

## Ethics Statement

Ethical review and approval was not required for the study on human participants in accordance with the local legislation and institutional requirements. The patients/participants provided their written informed consent to participate in this study.

## Author Contributions

GP and BP: formal analysis, investigation, software, and writing – original draft. RM and GS: validation, investigation, and supervision. FM: resources. FM and EJ: visualization. RdS and FM: funding acquisition. EJ: validation and data curation. JdC and RdS: conceptualization and supervision. JdC: formal analysis, methodology, and writing – original draft. RdS: writing – review and editing. All authors have read and agreed to the published version of the manuscript.

## Funding

This work is supported in part by CNPq – Brazilian National Research Council (No. PQ-2 312180/2019-5 on Cybersecurity No. 465741/2014-2), in part by the National Auditing Department of the Brazilian Health System SUS (No. DENASUS 23106.118410/2020-85), in part by the Brazilian Ministry of the Economy (Nos. DIPLA 005/2016 and ENAP 083/2016), in part by the Administrative Council for Economic Defense (No. CADE 08700.000047/2019-14), in part by the General Attorney of the Union (No. AGU 697.935/2019), and in part by the General Attorney's Office for the National Treasure (No. PGFN 23106.148934/2019-67).

## Conflict of Interest

The authors declare that the research was conducted in the absence of any commercial or financial relationships that could be construed as a potential conflict of interest.

## Publisher's Note

All claims expressed in this article are solely those of the authors and do not necessarily represent those of their affiliated organizations, or those of the publisher, the editors and the reviewers. Any product that may be evaluated in this article, or claim that may be made by its manufacturer, is not guaranteed or endorsed by the publisher.
